# Supplementary material for: An intrauterine cavity morcellator: A novel approach to high volume uterus morcellation. Ex-vivo study
Source: PLoS One. 2023 Mar 17;18(3):e0282149. doi: 10.1371/journal.pone.0282149 (PMC10022810; doi:10.1371/journal.pone.0282149)
Supplement: S1 File — (PDF) [file pone.0282149.s001.pdf]

Ex-Vivo Study of Heracure Device for Vaginal Intrauterine Cavity Morcellation

Protocol Number CP- 0300 Rev F

Protocol Date: Nov 17, 2022 (revised per PLUS ONE request)

PRINCIPAL INVESTIGATOR
Klein Zvi, M.D.
Head of Urogynecology Unit, Meir Medical Center, Kfar-Saba, Israel
(Phone: 972.9.7471820,Email: kleinz@clalit.org.il)

PI Signature: ______________________ Date: _______________________

SPONSOR
HERACURE Medical
Phone: 972.54.9011128
Email: galmeist1@gmail.com
Contact: Gal Meister, CEO

MONITOR
HERACURE Medical

Table of Contents

1. REVISION HISTORY 3
2. ACRONYMS, INITIALS AND DEFINITIONS 3
3. SYNOPSIS 4
4. INTRODUCTION 4
5. DEVICE DESCRIPTION 5
6. STUDY DESIGN 5
7. STUDY OBJECTIVES 6
8. STUDY END-POINTS 6
9. STUDY INCLUSION/EXCLUSION CRITERIA 6
10. STUDY PROCEDURE/FLOW 7
11. DURATION OF STUDY 9
12. RISK ANALYSIS 9
13. MANAGEMENT OF ADVERSE EVENTS 9
14. ETHICS 9
15. COMPENSATION 10
16. DEVIATION FROM PROTOCOL 10
17. SUSPENSION OF THE INVESTIGATION 10
18. PUBLICATION POLICY 11
19. ADMINISTRATIVE PROCEDURE OF THE INVESTIGATION 11
20. STATISTICAL ANALYSIS 11
21. CONFIDENTIALITY 11

1. REVISION HISTORY

Revision	ECO#	Date	Change Subject
A, B, C, D, E	< Refer to CP-0300 Rev E – Section 1 - "Revision History" >		
F	0033	17-Nov-2022	Revisions per PLUS ONE request

2. ACRONYMS, INITIALS, and DEFINITIONS

AE	Adverse Event
CBC	Complete Blood Count
CRF	Case Report Form
EC	Ethics Committee
GCP	Good Clinical Practice
IFU	Instruction for Use
LDH	Lactate Dehydrogenase
PAP	Papanicolaou
SAE	Serious Adverse Event

3. STUDY ABSTRACT/SYNOPSIS

**Protocol Title:**	Ex-Vivo Study of Heracure Device for Vaginal Uterine Intrauterine Cavity Morcellation
**Protocol No**:	CP-0300
**Sponsor:**	Heracure Medical Ltd.
**Study Objective:**	The purpose of this feasibility EX-VIVO study is to evaluate the safety and efficacy of the device for vaginal intrauterine cavity morcellation (uterine size reduction) after vaginal/laparoscopic hysterectomy
**Study Design:**	Observational Study, Single-arm
**Study Device:**	Heracure Device
**Number of Sites**	1
**Population**	Participants will be recruited from a list of patients consented for hysterectomy with uterus size ≤ 18 weeks. During the procedure, the Heracure System will be used for vaginal uterine size reduction, where the safety & efficacy of the device will be evaluated.
**No. of Subjects**	Up to 30
**Follow Up**	Not required
**Study Duration**	Up to 12 months
**Procedure:**	<u>EX-VIVO Feasibility Study on Extirpated Uteri</u><br>The purpose of this feasibility EX-VIVO study is to evaluate the safety and efficacy of the device for vaginal intrauterine cavity morcellation (uterine size reduction).
**Study Endpoints**	<u>Safety – Serious Adverse Device Effects</u><br>Procedure Safety will be assessed by recording all the adverse device effects that occur during the treatment with Heracure System.<br>Adverse Event for the ex-vivo study will be defined as uterine perforation at the end of procedure, assessed by inflating the uterus with saline.
**Study Secondary Endpoints**	<u>Device Success/ Physician satisfaction</u><br>Defined as ability to reduce uterus size/circumference, and the time required for performing the morcellation procedure

4. INTRODUCTION

<u>MORCELLATION OF LARGE UTERI</u>

Minimally invasive surgery is commonly used for hysterectomies because of its many benefits over open surgery. Uterine size is one of the major determining factors in selecting the ultimate hysterectomy surgical approach[1]. A measurement of approximately 12 weeks or less usually allows for a vaginal approach[1-4]. If the estimate of uterine size is larger than 12 weeks, it is likely that the surgeon will choose an abdominal surgical approach. If the uterine size is estimated above 18 weeks, it is almost certain that an abdominal approach will be selected. The abdominal approach is associated with longer hospital stay and recovery time, greater pain, and greater risk of infection, and minimally invasive approaches are associated with shorter lengths of stay and faster recovery times[2,6].

Although small uteri can be removed whole in this approach, larger specimens must be morcellated[1]. Since abdominal morcellation has become controversial recently because of concerns regarding dissemination of occult malignancy and since the current solution of abdominal bag morcellation is cumbersome and time consuming, new techniques that allow surgeons to offer women with large uteri a minimally invasive approach should be investigated and encouraged.

References:

1. Benassi L, Rossi T, Kaihura CT, et al. Abdominal or vaginal hysterectomy for enlarged uteri: a randomized clinical trial. Am J Obstet Gynecol 2002; 187: 1561–1565.
2. Choosing the route of hysterectomy for benign disease. ACOG Committee Opinion No. 4444. American College of Obstetricians and Gynecologists. Obstet Gynecol 2009; 114: 1156–1158.
3. Nieboer TE, Johnson N, Lethaby A, et al. Surgical approach to hysterectomy for benign gynecological disease. Cochrane Database Syst Rev 2009; 3: CD003677.
4. Varma R, Tahseen S, Lokugamage AU, et al. Vaginal route as the norm when planning hysterectomy for benign conditions: change in practice. Obstet Gynecol 2001; 97: 613–616.
5. Doucette RC, Sharp HT and Alder SC. Challenging generally accepted contraindications to vaginal hysterectomy. Am J Obstet Gynecol 2001; 184: 1386–1389; discussion 1390–1.

5. DEVICE DESCRIPTION

5.1 Device Identifications

Catalogue No.	Description	Manufacturer
HERA-RS-2000	Heracure System	Heracure Medical Ltd., Israel

Traceability is established through the use of Lot Numbers for each Finished Device lot.

5.2 Intended Use:

Heracure System is intended for use in gynecologic and general surgical endoscopic procedures by trained professional professionals in hospital environments and ambulatory surgery centers.

5.3 Device Description

Heracure System consists of the following components:

- Heracure Resection Device
- Motor Control Unit and Foot Pedal
- External Vacuum Source Device with Sterile Tubing Sets

The Heracure System features a rotating/oscillating cutting blade for resection of target tissue. The device's cutting blade is controlled by the controller. Distention/Irrigation fluid and resected tissue are transported from Heracure Resection Device to a vacuum canister via sterile disposable tubing-sets protruding from the proximal end of Heracure Resection Device.

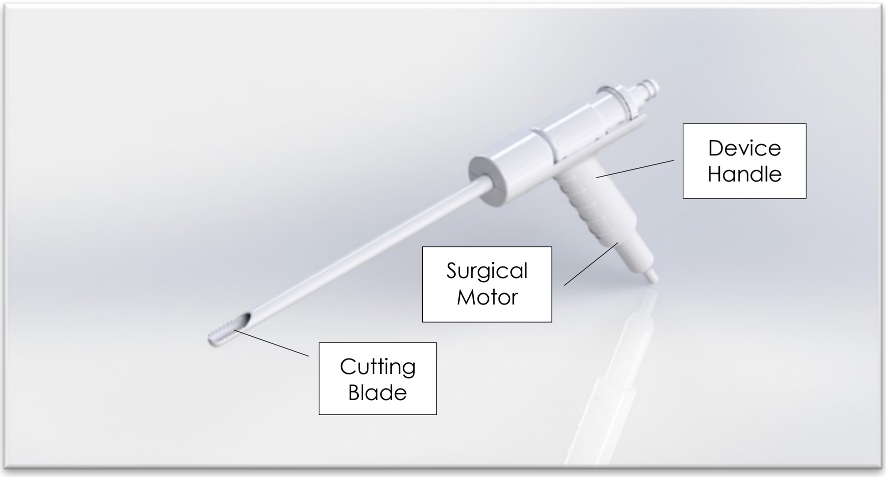

Figure 1: Heracure System

6. STUDY DESIGN

The study is designed as an observational study, single-arm study. Any patient who is scheduled for hysterectomy procedure that match the inclusion/exclusion criteria will be offered to give consent.

7. STUDY OBJECTIVES

The purpose of this feasibility EX-VIVO study is to evaluate the safety and efficacy of the device for vaginal intrauterine cavity morcellation (uterine size reduction) after vaginal/laparoscopic hysterectomy

8. STUDY ENDPOINTS

8.1 Primary Endpoints & Success Criteria

Primary Endpoint	Test Method
**Safety – Serious Adverse Device Effects**	Procedure Safety will be assessed by recording all the adverse device effects that occur during the treatment with Heracure System.<br>Adverse Event for the ex-vivo study will be defined as uterine perforation at the end of procedure, assessed by inflating the uterus with saline.

8.2 Secondary Endpoints & Success Criteria

Secondary Endpoint	Test Method
**Device Success/ Physician Satisfactory**	Device Success is defined as ability to reduce uterus size/circumference, and the time required for performing the morcellation procedure

9. STUDY INCLUSING/EXCLUSION CRITERIA

9.1 Inclusion Criteria

- Healthy female aged above 21
- Scheduled for hysterectomy
- Subject has a normal Papanicolaou test (PAP smear)
- Subject signs and dates a written informed consent form (ICF) and indicate an understanding of the study procedures.

9.2 Exclusion Criteria

- Subject is pregnant
- Subject is suspected to have endometrial pathology, such as hyperplasia or carcinoma (abnormal Pipelle result or ultrasound screening)
- Subject is suspected to have cervical cancer (abnormal PAP smear result)
- Subject is suspected to have leiomyosarcomas prior to surgery (abnormal signs of rapid growth of lesion, LDH levels or ultrasound screening)
- Subject is suspected to have HIV infection, hepatitis B, CJD, or other sexually transmitted diseases or illness.
- Any other reason for which the individual study subject is not appropriate or suitable for participation
- Subject is unable to give consent

10. STUDY PROCEDURE/FLOW

10.1 Study Procedure for EX-VIVO Study

ASSESSMENT	SCREENING/ PRE-PROCEDURE	PROCEDURE	DISCHARGE	FOLLOW-UP VISIT 30 DAYS
Inclusion/Exclusion Criteria	X			
Cervical Screening: PAP Smear Test	X			
Endometrial biopsy (Pipelle) - in case of irregular bleeding	X*			
Laboratory tests: Lactate Dehydrogenase (LDH) test	X*			
Informed Consent	X			

- If applicable

10.2 Study Phases

10.2.1 <u>Screening and Enrollment/Pre-procedure</u>

10.2.1.1 The physician will allocate appropriate candidates for enrollment based on meeting the general inclusion criteria.

10.2.1.2 If the patient is interested in participating in the study, following a thorough explanation from the physician, the patient will sign an informed consent form.

10.2.1.3 The physician will then verify that the patient complies with the study inclusion/exclusion criteria.

10.2.1.4 Endometrial biopsy (Pipelle) will be used to rule out endometrial pathology, such as hyperplasia or carcinoma. PAP smear test is used to rule out cervical cancer. Lactate Dehydrogenase (LDH) test and the ultrasound screening will be used to rule out patients suspected to have leiomyosarcomas. If cancer is suspected, patient shall be excluded/removed from the study.

10.2.2 <u>Procedure for Conducting EX-VIVO Study on Extirpated Uteri</u>

10.2.2.1 The Study will be conducted on whole extirpated uterus.

10.2.2.2 In order to minimize the interference with the hospital routine pathology analysis of uterus, an endometrial biopsy ("Pipelle") will be performed prior to the hysterectomy procedure to enable the data for ruling out endometrial pathology, such as hyperplasia or carcinoma.

10.2.2.3 The purpose of this feasibility EX-VIVO study is to evaluate the safety and efficacy of the device for intrauterine cavity morcellation (e.g., reducing uterine size from the inside of the uterine cavity). During the morcellation procedure, a protective barrier will be wrapped around the uterus.

10.2.2.4 The size of the uterus (e.g., circumference, weight) shall be recorded before and after the morcellation procedure, as well as the total resection time.

10.2.2.5 To assess uterine perforation at the end of procedure, the uterus shall be inflated with saline and observed for leakage.

10.2.2.6 At the end of the procedure, the entire uterus and its resected tissue will be sent to pathology for hospital routine pathology analysis.

11. DURATION OF STUDY

Study duration up to 12 months

12. RISK ANALYSIS

The risks related to the investigational procedure are mitigated by:

12.1 Inclusion/Exclusion Criteria, which are provided in Section 9 of this protocol.

12.2 Consistent with the current hospital protocol, after completing the testing, all samples will be returned to pathology, where routine pathological analysis will be conducted. In the case that the pathologist on site is identifying an abnormal appearance of the uterine tissue, study will be discontinued, and samples will be sent to pathology, where routine pathological analysis will be conducted.

13. MANAGEMENT OF ADVERSE EVENTS

13.1 An adverse event is any undesirable or unintentional event that occurs during the course of the study, considered related to Heracure device. Regardless of severity or relationship to the investigational device, all adverse events occurring during the study must be recorded in the patient's CRF.

13.2 Due to the nature of the current study performed on ex-vivo extirpated uterus (no patient is involved), an adverse event will be defined in the study as uterine perforation at the end of procedure, assessed by inflating the uterus with saline, or any event that leads to interference with the hospital routine pathology analysis of the uterus post procedure.

14. ETHICS

14.1 Declaration of Helsinki

The study will be conducted according to the guidelines established in the Declaration of Helsinki, ICH E-6 GCP Guidelines and ISO 14155 requirements.

14.2 Patient Withdrawal from the Study

14.2.1 Reasons for removing a patient from the ex-vivo study

- Patients who express a desire to withdraw from the study.
- Any medical condition that, in the opinion of the investigator, warrants discontinuation from the study.
- In the case that the pathologist on site is identifying an abnormal appearance of the uterine tissue, study will be discontinued, and samples will be sent to pathology.

14.2.2 The investigator must record the reason for any patient withdrawal from the study.

14.2.3 Patients withdrawn for any reason will be replaced by patients who meet the inclusion and exclusion criteria at the discretion of the principal investigator.

15. COMPENSATION

Compensation will not be provided to the participants.

16. DEVIATIONS FROM PROTOCOL:

16.1 The study will be conducted in compliance with the protocol, ICH-GCP guidelines, and the applicable regulatory requirements.

16.2 A protocol deviation is defined as any event occurring in a study, which is not in compliance with the requirements of the protocol and for which an amendment has not been granted. The Investigator and the study staff will not deviate from the protocol without prior written approval from the Sponsor.

16.3 When circumstances arise which suggest that a deviation from this protocol should be considered, the investigator or other physician in attendance must contact the study monitor by telephone as soon as possible prior to implementation. Any deviation from protocol agreed to will pertain only to the individual patient/subject involved. The case report form will describe the circumstances and identify the pertinent protocol procedure.

16.4 Significant deviations defined as compromising or potentially compromising the safety of the patients, enrolment of non-eligible patients and any deviation which compromises significantly the outcome of the study, shall be subject to reporting to the EC within the appropriate deadlines indicated by the EC.

16.5 In the event that a protocol change is proposed for all patients, an amendment will be submitted to the IRB. The protocol change (amendment) must be approved by the IRB prior to being initiated.

17. SUSPENSION OF THE INVESTIGATION

17.1 The investigation may be suspended or terminated in the event of human injury as a result of direct failure of the device.

17.2 The sponsor will report the SAE to the EC / Competent Authority as required. The Principal investigator and company medical advisors will evaluate the case and consider whether the study may continue, requires modification and amendments or should be terminated.

18. PUBLICATION POLICY

18.1 Heracure Medical recognizes that the availability of study results is important to the communication of significant new information for the medical profession, patients and the general public. Such communication includes publication of a paper in a peer-reviewed medical journal, abstract submission with a poster or oral presentation at a scientific meeting, or making study results public by some other means.

18.2 Heracure Medical designs and conducts, together with the Investigators, studies in an ethical and scientifically rigorous manner to determine the benefits, risks, and value of medical device products. As Sponsor of such studies, Heracure Medical receives from the Investigators at all research sites, and subsequently verifies, the data for the studies it conducts. As owners of that data, Heracure Medical works diligently with the Investigators to ensure the accuracy and integrity of the entire study database.

19. ADMINISTRATIVE PROCEDURES OF THE INVESTIGATION:

Investigator sites and study documentation may be subject to Quality Assurance audits during the course of the study. In addition, inspections may be conducted by regulatory bodies at their discretion, during and after study completion.

20. STATISTICAL ANALYSIS

Descriptive statistics will be provided.

21. CONFIDENTIALITY

The subject’s name and personal data will remain confidential and will not be published in any way. Patients will be identified on all CRFs by a unique reference number. CRFs are confidential documents. The sponsor’s monitor or representative and regulatory representatives, auditors and inspectors may have access to medical files in order to verify authenticity of data collected. Accordingly, data will only be available to the sponsor, the Investigator, Investigators’ staff, the investigation statistician, and if requested to the advisory committee and regulatory authorities.

The Investigator will maintain as part of the investigation file a list identifying all patients entered into the study.
