# Supplementary material for: An intrauterine cavity morcellator: A novel approach to high volume uterus morcellation. Ex-vivo study
Source: PLoS One. 2023 Mar 17;18(3):e0282149. doi: 10.1371/journal.pone.0282149 (PMC10022810; doi:10.1371/journal.pone.0282149)
Supplement: Attachment — Submitted filename: EDIT_Response to reviewers 28.11.docx [file pone.0282149.s002.docx]

28.11.2022

Dr. Andrea Giannini

Academic Editor

PLOS ONE

Re: Manuscript ID PONE-D-22-26314

An Intrauterine Cavity Morcellator: A Novel Approach to High Volume Uterus Morcellation. EX-VIVO Study

Dear Dr.Giannini

Thank you for your letter regarding our above-cited manuscript. We would also like to thank the reviewers for their thoughtful comments and appropriate suggestions. We have revised the manuscript extensively, carefully addressing all the comments and suggestions. The changes made in the revised manuscript are indicated by “highlight function" in Word. Our point-by-point responses to all the comments are listed below.

We hope you will now find this manuscript suitable for publication in the PLOS ONE journal.

Thank you for your consideration.

On behalf of all authors,

Yair Daykan, MD

Corresponding author

Reviewer(s)' Comments to Author:

Journal Requirements:

**Reviewer #1:**

1. There are a few spelling errors needs to be corrected

- Spelling errors and English grammar were corrected

2. The author mentions use of t test and p value in the methodology whereas there has been no such analysis in the results section

- The result section has been corrected.

**Reviewer #2:**

1. The Authors did not mention the sample size calculation for their study. It is essential to specify this data in order to guarantee an adequate significance of the results obtained by the Authors.

- Thank you for your question. The study's primary endpoint was to evaluate the feasibility and safety of novel morcellation equipment. Though this was a small-scale pilot study, we couldn’t calculate a sample size for this trial as we don’t have the frequency of perforation to such an intrauterine device. In our subsequent trial, which will be a larger cohort, we will calculate a sample size according to the primary outcome

2. The authors have not adequately highlighted the strengths and limitations of their study. I suggest better specifying these points.

- Thank you, we elaborate on these at the end of the discussion section.
- Line 273
- " The main strength of this first preliminary report is its novelty of demonstrating the feasibility and safety of a new intrauterine approach to perform morcellation for uterine size reduction. In our opinion, safety, the most critical point at this stage was demonstrated by the ability to avoid perforation. The Heracure device will enable the use of a minimally invasive approach (laparoscopic and vaginal) for hysterectomy in cases that currently are feasible only with vaginal manual morcellation or need to increase the abdominal access due to the size of the uterus. "

3. What are the actual clinical implications of this study? it is important to report the results obtained by the authors in the context of clinical practice and to adequately highlight what contribution this study adds to the literature already existing on the topic and to future study perspectives.

- We elaborate on this point in the discussion section.
- Line 263
- "This trial demonstrates feasibility of a novel morcellation approach, which can theoretically solve the current day-to-day difficulties in the extraction of large uteruses, during a minimally invasive approach for hysterectomy, by introducing a new intrauterine morcellation device. This unique approach enables the extraction of the uterus, even in cases of large myomas that currently preclude the use of the laparoscopic and the vaginal approach morcellation [1,4,5]. The potential clinical advantage of intrauterine morcellation is avoiding the need for manual vaginal morcellation and its potential risks. Moreover, this tool may reduce the risk of intraperitoneal spreading of potentially malignant cells as well as overcome some technical difficulties and improve cosmetic results of the traditional approach for extraction of the uterus."

4. Does this manuscript conform the Enhancing the Quality and Transparency Of health Research (EQUATOR) network guidelines? It would be mandatory to declare about this element.

- We declare this element in the methods section.
- Line 96
- " This study used the implementation of enhancing the Quality and Transparency Of health Research (EQUATOR) network guidelines."

**Reviewer #3:**

1. Does Heracure morcellator stop automatically or with surgeon’s manipulation?

- We elaborate on this important point in the text.
- Line 128
- " In the future during the in-vivo procedure, a proximity magnetic sensor located inside the protective barrier will automatically stop the morcellation motor when the morcellator knives are close to the barrier, to prevent any iatrogenic damage. A laparoscopic camera will accompany the procedure to verify safety and prevent potential visceral injury."

2. In general, the uterus, which is too large to remove through vaginal orifice is a good indication for morcellation. However, the uterus removed by TAH (case5, 9and 10) tend to suggest poorer response for morcellation and longer time to spend it. Only from the data suggested in this manuscript, it is uncertain whether Heracure morcellator is more useful compared to other kinds of hysteroscopic morcellators.

- Thank you for this important question. The TAH morcellation cases had enlarged uteruses with a mean size of 18w and a mean resection time of 8.8 minutes. In this preliminary study, we aim to assess the feasibility of this device and included only three cases of such large uteri. Future in-vivo trials will involve a higher number of patients with large uteri to approve its feasibility and superiority over the traditional approaches. Moreover, the goal of this new intrauterine device is morcellation for uterine size reduction during hysterectomy. As far as we know, the use of current hysteroscopic morcellators are for intrauterine pathologies when uterine preservation is needed. As these are two different goals of treatment they are not comparable.
- We added this clarification to the limitation section.
- Line 282
- " The current trial has several limitations. The main limitation was its small cohort size that included only three cases of larger uteri with a mean size of 18w and mean resection time of 8.8 minutes. To further assess whether this device can be effective in such cases, future in-vivo trials, will involve a higher number of patients with large uteri, to approve its feasibility and superiority over the traditional approaches."

**Reviewer #5:**

1. In the material and method section Page 10 Lines 84 & 85, you mentioned that Patients were followed-up for 6 weeks post – operative. What is the indication for postoperative follow-up while your technique was done ex-vivo on extirpated uteri? Especially that No intervention with your new technique were applied in the patients. And you mentioned that in the lines 87&88 that (No intervention was applied in the patients, as the study only investigated the post-hysterectomy uterus.)

- Thank you for this comment, we accept it, and since this study investigates only the post-hysterectomy uterus, we omitted this sentence.

2. In the page 10 line 90 you mentioned that the primary outcome evaluated was the procedure’s safety, how could be determined by this small number of patients (10 patients) and the ex-vivo procedure and choosing only uteri with benign lesions.

- In every innovative medical tool, it is accepted to evaluate first using a small number of patients to prove the concept and safety. In our opinion, safety was the most critical point at this stage and was expressed by the ability to avoid perforation. We were not aiming for cases with malignancies (part of the exclusion criteria) and only focused on benign lesions such as myomas or cases of prolapsed uteri. Usually, those pathologies rarely involve malignancies. Furthermore, intrauterine morcellation reduces uterine size without exposure of morcellated tissue to the abdominal cavity.
- Line 282
- " The current trial has several limitations. The main limitation was its small cohort size…

An additional limitation is the trial excluded cases with pathologies that involve malignancies. Of note, the current approach, intrauterine morcellation, as opposed to intraperitoneal morcellation, reduces the uterine size without exposure of morcellated tissue into the abdominal cavity. "

3. In the case selection section, page 11, you selected only uteri with benign lesions, you ought to utilize uteri with benign and malignant lesions where the liability for perforation of uterine walls and spreading of malignant cells is more with malignant uterine lesions where the point of your review is to test the safety of the technique in keeping away from perforating the uterine walls and expected spread of malignant cells or injury to nearby vital organs.

The protective barrier that was folded over the uterus and was utilized for the separation and avoidance of potential intraperitoneal visceral injury is opened at its upper end, so won't forestall the spillage to the peritoneal cavity assuming perforation of uterine wall happened especially with the utilization of irrigation fluid in controversy to the use of in bag power morcellation that were shown in previous studies.What is the material and length and flexibility of the barrier used and the method of its intraoperative application to surround the whole uterus especially if it is large volume (as 20 weeks size) with interstitial or subserous myomata?

- Thank you for this question- the barrier is flexible and arrives in different sizes to adjust different sizes of uterine. We elaborate further on these important points in the text.
- Line 128
- " In the future, during the in-vivo procedure, a proximity magnetic sensor located inside the protective barrier will automatically stop the morcellation motor when the morcellator knives are close to the barrier, to prevent any iatrogenic damage. A laparoscopic camera will accompany the procedure to verify safety and prevent potential visceral injury."

4. In the case number 3& 5&9 &10 the uterine size was large with fibroids what is the type of it, subserous, submucous or interstitial in each case.

- In all the myoma cases, we had type 1-5 myoma (FIGO classification.)
- Case 3- Type 4
- Case 5- Type 1,3,5
- Case 9- Type 5
- Case 10-Type 4-5

5. In case number 1 what is meant by the indication of risk reduction in a uterus weight 365 gram. What is the pathological diagnosis before and after hysterectomy of this case?

- This woman had Lynch syndrome- It was mentioned in Table 2

8. As the authors mentioned, (in page 19 line 290), In-VIVO trials are required to strength these finding of this limited observation cohort for the safety and efficacy of the procedure, especially that the application of the protective barrier might limit the manipulation of the uterus and visualization of the surrounding bladder or intestine.The chance of uterine perforation won't diminish with this maneuver since morcellation is done indiscriminately without hysteroscopic guide and the role of laparoscopy is just to screen in the event that perforation happened or not and to protect intestine or bladder from injury.If the uterus enlarged by huge subserous myoma uterine perforation definitely occurs and possibility of dissemination of malignant cells is high than enclosed abdominal morcellation.

- The barrier is flexible and arrives in different sizes to adjust different sizes of uterine. In the future, during the in-vivo procedure, a proximity magnetic sensor located inside the protective barrier will automatically stop the morcellation motor when the morcellator knives are close to the barrier, to prevent any iatrogenic damage.

**Reviewer #6:**

Introduction

1. Line 5 rapid recovery [1–3], the cited reference in text to be spaced out from the word. This applies throughout the manuscript.

- Thank you for this point- we corrected this throughout the manuscript.

Materials and Methods

2. Line 87 & Line 130, Ex VIVO is to be written as Ex vivo.
3. Line 136 & Line 290, In VIVO is to be replaced with In vivo

- This change was modified throughout the manuscript.

Results

4. Table 1 range is to be denoted as min – max values in the table footnote. Percentage figures to be at least 1 decimal point. Sample size is to be indicated in the table. The word Data Mean Range is to be removed and replaced with Variables. Pre-resection uterine size or uterine weight? Size and weight are different in meaning. The short form for gram is g.

- Thank you for this comment; these points were modified in the table

5. Table 1 & 2, decimal points for minutes to be added. Was the resection time captured in minutes converted from total seconds?

- It was added. The time taken in a sec and converted to min.
- For example: 250 sec /60=4.1 min

6. Table 2, columns and data for post-resection uterus size and post-resection uterus circumference to be added before deriving uterus weight and circumference reduction data. Percentage figures to be at least 1 decimal point.

- We added it to the table.

7. Statistical analyses were stated in the manuscript, but there was no statistical analysis output presented. If there were no statistical tests performed, the writeup on statistical analyses is to be omitted, and the reason is to be stated in the method section.

- We corrected the statistic section.

8. Line 215, RR is to be spelled out when first used

- Was added to the text
- Line 208

Discussion

9. Line 216, proper unit is to be presented for cm2 e.g cm ^2 (superscript 2).

- Was corrected in the text
- Line 210

10. Some page numbers which were provided in the TREND Checklist did not tally with the page number in the manuscript. If there is no information provided in the manuscript, the page number should not to be written in the checklist. A write-up could be provided to mention those information were not relevant/applicable in the manuscript

- The TREND Checklist was corrected. Places without information in the manuscript write-up as NA
