# Supplementary material for: An intrauterine cavity morcellator: A novel approach to high volume uterus morcellation. Ex-vivo study
Source: PLoS One. 2023 Mar 17;18(3):e0282149. doi: 10.1371/journal.pone.0282149 (PMC10022810; doi:10.1371/journal.pone.0282149)
Supplement: Attachment — Submitted filename: Response to reviewers 15.1.23.docx [file pone.0282149.s003.docx]

15.1.2023

Dr. Andrea Giannini

Academic Editor

PLOS ONE

Re: PONE-D-22-26314R1

An Intrauterine Cavity Morcellator: A Novel Approach to High Volume Uterus Morcellation. EX-VIVO Study

Dear Dr.Giannini

Thank you for your letter regarding our above-cited manuscript. We would also like to thank the reviewers for their thoughtful comments and appropriate suggestions. We have revised the manuscript extensively, carefully addressing all the comments and suggestions. The changes made in the revised manuscript are indicated by “highlight function" in Word. Our point-by-point responses to all the comments are listed below.

We hope you will now find this manuscript suitable for publication in the PLOS ONE journal.

Thank you for your consideration.

On behalf of all authors,

Yair Daykan, MD

Corresponding author

Reviewer(s)' Comments to Author:

Journal Requirements:

**Reviewer #3**:

Thank you for resubmitting your manuscript.

As I mentioned previously, the entry number is too small. It seems a kind of preliminary data with supporting Heracure Medical Ltd. It must not reach to the level of this journal.

According to your result, the indication to apply Heracure device seems rather small. The enclosed in-bag abdominal morcellation is more useful to remove larger-sized uterus.

You need to analyze not only the weight and circumference of the uterus but also the shape and the level of flexion to regulate the appropriate indication.

In addition, I am wondering whether the radial panel completely protect the dissemination of the small component of the removing uterus. Compare with the protection you suggested in the revised manuscript, in-bag morcellation seems rather simple and safe. I hope you to submit the renewal manuscript with additional information after further investigation.

- Dear reviewer,

Thank you for these comments. The purpose of this manuscript is to present the preliminary experience with a new device in an ex-vivo study. In this preliminary study manuscript, we demonstrate the feasibility of the Heracure device.

At the initial investigation of every innovative medical tool, it is acceptable to first evaluate proof of concept and safety in a small number of patients. Our subsequent trial in-vivo, will include a larger cohort, in order to demonstrate the advantages of such a tool and highlight the safety with a new added on component to the flexible protective barrier and the proximity magnetic sensor.

We are sure that the readers will also benefit from this publication, despite its small cohort.

Nevertheless, I would like to draw your attention to previous publications that similarly began to investigate an innovative tool like our tool, in a small cohort. For this example, I choose the vNOTES approach.

| Source, year | Study type | No. of patients | Main surgery type |
| --- | --- | --- | --- |
| Lee et al, ^25^  2015 | Case series | 4 | Robot-assisted hysterectomy |
| Wang et al, ^18^  2016 | Retrospective matched case–control | 34 | NOTES-assisted ovarian cystectomy |
| Baekelandt, ^23^  2015, Baekelandt, ^24^  2016 | Case series | 10 | Hysterectomy |
| Baekelandt, ^29^  2015 | Video report | 1 | Adnexectomy |
| Lee et al, ^26^  2014 | Case series | 3 | Myomectomy |
| Lee et al, ^27^  2014 | Case series | 3 | NOTES-assisted hysterectomy + salpingo-oophorectomy + pelvic lymphadenectomy |
| Xu et al,  ^17^ 2014 | Prospective randomized | 18 | Salpingectomy (pure NOTES = 17, hybrid NOTES = 1) |
| Yang et al, ^21^  2014 | Retrospective matched case– control | 16 | NOTES-assisted hysterectomy |
| Yang et al, ^16^  2013 | Case series | 7 | Salpingo-oophorectomy(  *n* = 3), oophorectomy (  *n* = 3), paraovarian cystectomy(  *n*  = 1) |
| Lee et al, ^28^  2012 | Case series | 15 | Tubal sterilization (  *n*  = 2), salpingectomy (  *n* = 2), ovarian tumor enucleation(  *n*  = 1), NOTES-assisted hysterectomy (  *n* = 10) |
| Su et al,  ^19^ 2012 | Case series | 16 | NOTES-assisted hysterectomy |
| Ahn et al, ^14^  2012, Kim,  ^15^ 2013 | Case series | 10 | Oophorectomy (  *n* = 3), salpingostomy (  *n* = 2), salpingectomy (  *n* = 2), ovarian cystectomy (  *n*  = 1), paratubal cystectomy(  *n*  = 1), ovarian wedge resection (  *n*  = 1) |
| Lee et al, ^13^  2012 | Case series | 10 | Tubal sterilization (  *n*  = 3), salpingectomy (  *n* = 3), ovarian tumor enucleation(  *n*  = 4) |

Yoshiki N. Review of transvaginal natural orifice transluminal endoscopic surgery in gynecology. Gynecol Minim Invasive Ther. 2017 Jan-Mar;6(1):1-5. doi: 10.1016/j.gmit.2016.11.007. Epub 2017 Jan 20. PMID: 30254860; PMCID: PMC6113962.

- We decided to analyze the circumference of the uterus and its weight since in our experience the uterine circumference is a major predictor for successful retrieval of the uterus through the vagina without opening and cutting of the uterus body.

**Reviewer #4:** Dear Author, the article has been edited in line with the suggestions. I think the article can be published in its current form.

**Reviewer #7:**

1. In the discussion, it would be appropriate to include the usual problem of morcellation with electrical instrumentation, versus morcellation with the scalpel. The electric morcellator involves mass being reduced by morcellation of tissue which is delivered to the pathologist as a meat "hamburger". The pathologist, unfortunately, is often unable to distinguish the anatomy to be analyzed and to indicate exactly the parts to be analyzed in anatomical reference of the bowel, given that he has no points of reference (PMID: 36292534). Conversely, manual morcellation with the scalpel allows the organ or mass to be removed and reconstructed with a good approximation using sutures. Therefore, I am convinced that this specification can be useful to readers of such a complex and controversial topic.

- We thank your important input; we added these points and the reference into the discussion section.
- Line 241
- " When comparing the electrical instrumentation versus morcellation with the scalpel, we can reveal one of the main limitations of the current in-bag morcellation concerning the pathology examination. The electric morcellator involves a mass being reduced, delivered to the pathologist as a slice of ground tissue. Unfortunately, the pathologist is often unable to distinguish the anatomy to be analyzed and to indicate exactly the parts to be analyzed, given that there are no reference points [28]. Conversely, manual morcellation with the scalpel allows the organ or mass to be removed and reconstructed with a good approximation using sutures. This novel tool overcomes this disadvantage providing both the anatomy preservation such as in cold knife morcellation and the efficacy of the electrical morcellation."

**Reviewer #8:** I read with great interest the manuscript, which falls within the aim of this Journal. In my honest opinion, the topic is interesting enough to attract the readers’ attention. Nevertheless, the authors should clarify some points and improve the discussion, as suggested below.

Authors should consider the following recommendations:

1. Manuscript should be further revised in order to correct some typos and improve style.

- We thank the reviewer for the important remark, and we corrected accordantly throughout the manuscript.

2. Authors should add further elements to discuss the role and technical differences among Intrauterine Cavity Morcellator (authors may refer to: PMID: 28948169), highlighting that these devices were not affected by the FDA warning about morcellation, which led to the adoption of endobag use as mandatory to avoid the dissemination of misdiagnosed uterine sarcomas (authors may discuss this point referring to: PMID: 35263843; PMID: 36553227).

- Thank you, we added these points into the manuscript.
- Line 267
- " Intrauterine morcellation is also familiar in the hysteroscopy field. The hysteroscopic tissue removal system seems a feasible surgical option regarding operative time and complications[34]. This hysteroscopic intrauterine morcellation was not affected by the FDA warning about morcellation in relation to the possible dissemination of misdiagnosed uterine sarcomas[35,36]. Unfortunately, the type and size of the myoma remain the biggest challenge in such a technique."

3. I recommend to highlight novel pieces of evidence about the minimally invasive management of hysterectomy for large uteri and/or for different indications, as well as the surgical specimen extraction (authors may refer to: PMID: 36498515; PMID: 34730067).

- Thank you, we added these points into the manuscript.
- Line 49- reference 4, Line 54
- " and performed with the help of the in-bag (contained) morcellation[10]."
